# Differential rotational movement and symmetry values of the thoracolumbosacral region in high-level dressage horses when trotting

**DOI:** 10.1371/journal.pone.0251144

**Published:** 2021-05-06

**Authors:** Russell MacKechnie-Guire, Thilo Pfau

**Affiliations:** 1 Centaur Biomechanics, Moreton Morrell, Warwickshire, United Kingdom; 2 Department of Clinical Science and Services, The Royal Veterinary College, Hatfield, United Kingdom; Massey University, NEW ZEALAND

## Abstract

High-level dressage horses regularly perform advanced movements, requiring coordination and force transmission between front and hind limbs across the thoracolumbosacral region. This study aimed at quantifying kinematic differences in dressage horses when ridden in sitting trot–i.e. with additional load applied in the thoracolumbar region–compared with trotting in-hand. Inertial sensors were glued on to the midline of the thoracic (T) and lumbar (L) spine at T5, T13, T18, L3 and middle of the left and right tubera sacrale of ten elite dressage horses (Mean±SD), age 11±1 years, height 1.70±0.10m and body mass 600±24kg; first trotted in-hand, then ridden in sitting trot on an arena surface by four Grand Prix dressage riders. Straight-line motion cycles were analysed using a general linear model (random factor: horse; fixed factor: exercise condition; covariate: stride time, Bonferroni post hoc correction: P<0.05). Differential roll, pitch and yaw angles between adjacent sensors were calculated. In sitting trot, compared to trotting in-hand, there was increased pitch (mean±S.D), (in-hand, 3.9 (0.5°, sitting trot 6.3 (0.3°, P = <0.0001), roll (in-hand, 7.7 (1.1°, sitting trot 11.6 (0.9°, P = 0.003) and heading values (in-hand, 4.2 (0.8), sitting trot 9.5 (0.6°, P = <0.0001) in the caudal thoracic and lumbar region (T18-L3) and a decrease in heading values (in-hand, 7.1 (0.5°, sitting trot 5.2 (0.3°, P = 0.01) in the cranial thoracic region (T5-T13). Kinematics of the caudal thoracic and lumbar spine are influenced by the rider when in sitting trot, whilst lateral bending is reduced in the cranial thoracic region. This biomechanical difference with the addition of a rider, emphasises the importance of observing horses during ridden exercise, when assessing them as part of a loss of performance assessment.

## 1 Introduction

The vertebral column plays an important role for athletic performance, enabling the transfer of forces between the pelvic and thoracic limbs [1]. Across gaits and exercises, the vertebral column shows complex and varying movements, generally consisting of three rotational movements, categorized into flexion-extension, lateral bending and axial rotation, and three translational movements, categorized into dorsoventral, mediolateral and craniocaudal translation [2–5]. Movements of the vertebral segments differ–in relation to amplitudes and distribution between the different rotational components–between walk [5], trot [3] and canter [4] and spinal kinematics are influenced by limb movements [3–5] and show adaptations to unilateral forelimb [6] and hindlimb lameness [7].

The angulation and shape of the articular processes (facet joints) reflect the limited range of motion observed between adjacent vertebrae. In the horse, the thoracolumbar spine appears to be fairly rigid (or stiff: i.e. showing comparatively small amounts of movement for a given force) [8]. Despite its ‘rigidity’, the horse is able to modulate the amount of intervertebral movement through the surrounding soft tissues, the tone of the epaxial musculature and the elasticity of the interspinous and intervertebral ligaments [8]. It appears likely, that the comparatively smaller range of motion of the equine spine in comparison to smaller quadrupeds, is a sign of prioritizing stability for efficient force production over increased movement and energy storage. The addition of tack and rider further emphasises the need for stability to support the added weight [8].

In general, there is a paucity of quantitative evidence documenting the movement of the thoracolumbosacral spine in terms of the three rotations: flexion-extension, lateral bending and axial rotation in ridden exercise. It appears logical to hypothesise that the horse dynamically modulates spinal stiffness in relation to the varying degrees of stability (for force transmission) and energy storage (for efficient movement) in relation to the task at hand. This may vary across the vertebral segments and in particular may require increased ‘stability’ in the thoracolumbar region to support the additional weight of saddle and rider and to deal with the dynamic forces of the rider relative to the horse.

Horses have to manage dynamic influences, not only from the static mass of the rider, but also the dynamic forces of the horse-rider coupling [9]. In ridden trot exercise, the rider can adopt various riding positions (rising trot, sitting trot and two-point), which, depending on the chosen position, may influence head and pelvic symmetry parameters [10]. The presence of the rider, regardless of their position (sitting or rising), decreases the dorsoventral movement of the spine [11]. In rising trot, as a consequence of the rider raising their centre of mass into a standing position and then transferring their mass into a seated position within one motion cycle, the horse experiences an uneven biphasic load. This affects motion symmetry of the horse’s pelvis [12], in comparison to symmetrical riding positions (sitting trot and two-point) [10]. However, to the authors’ knowledge, symmetry values of the thoracolumbosacral region have not yet been reported during ridden exercise.

In contrast to movement symmetry, more quantitative evidence is available describing the rotational movements of the back and the influence of the rider. In trot, rotational movements of the back are influenced by the rider and the adopted position. Lateral bending of the lumbar spine increases its range of motion when the horse is being ridden in rising trot [13] compared to sitting trot. When the rider remains seated throughout the trot motion cycle (sitting trot), an overall extending effect on the back has been reported in comparison with rising trot [13]. However, due to marker occlusion by tack (saddle) and rider, [13] the kinematics of the cranial thoracic spine are underexplored. In order to provide dynamic stability in the presence of rider dynamics and their adopted riding position, it may be hypothesised that in sitting trot, the adjacent vertebral segments (cranial-mid thoracic) will alter their range of motion [14]. It is interesting to note that in rising trot, compared to in-hand, an increase in flexion-extension is measurable during the standing phase. During the seated phase however, different adaptations of spinal movement are observed in the cranial region to the caudal thoracic region (cranial: increase; caudal: decrease) [15]; the differences of flexion-extension in the cranial and caudal region support the idea that the equine back can alter its range of motion segmentally which could be an indication of dynamic stability. To the authors’ knowledge, rotational values of the thoracolumbosacral region have not been reported whilst horses are ridden in sitting trot.

The aim of the present study was to quantify kinematics of the thoracolumbosacral spine in elite dressage horses when ridden in sitting trot, in comparison to the unloaded state during trot in-hand without a rider. We hypothesise that, when ridden in sitting trot compared with an unloaded condition during trot in-hand, there will be: 1) increased range of motion of the caudal thoracic and lumbar region, 2) increased range of motion of the cranial thoracic spine and 3) no change in movement asymmetry parameters.

## 2 Materials and methods

This study was approved by the Royal Veterinary College ethics and welfare committee, project number URN 20181785–2. Informed, written consent was obtained prior to participation in the study. At the time of the study, all riders were free from any injuries and could withdraw their participation from the study at any point.

### 2.1 Horses

Ten elite dressage horses (competing at Intermediate I and above, FEI) were used in this study, ridden by their own rider. The horses were housed at two different facilities and were all part of an extensive equine sports science and medicine programme, including regular therapy and veterinary assessments. On the day preceding data collection, all horses were assessed by their respective veterinarian–this assessment included visual observations in walk and trot in a straight line on a firm level surface, as well as flexion tests of all four limbs; no lameness was observed. On the day of data collection, the horses underwent a physiotherapy examination, assessing the presence or absence of epaxial hypertonicity and pain. In addition, the horses’ gait asymmetry was quantified using a validated sensor system [16]. Eight geldings and two mares, with an average (+/- standard deviation) value for height at the withers of 1.70±0.10m, body mass of 600±24kg and age 11±1 years were recruited.

### 2.2 Riders

Two male and two female Grand Prix Dressage riders (FEI ranked) were recruited with an average (+/- standard deviation) height 1.82±0.08m and body mass 74±1kg. The riders trained and competed their respective horses used in the study.

### 2.3 Saddle and girth

Horses were ridden in their usual saddle, girth and bridle. Static and dynamic saddle fit was assessed independently by five Society of Master Saddlers Qualified Saddle Fitters (SMSQSF). Saddle details have been described elsewhere [17]. In brief, all horses were ridden in a plastic moulded treed, mono flap dressage saddle, featuring a panel which was lined with a pressure reducing material and flocked with wool (Fairfax Saddles, Walsall). Seat size remained the same and the stirrup length which the rider was accustomed to was used throughout. A high withered saddle cloth (H:58 cm withers to base, 54 cm lowest point to base of cloth x W:63 cm) was positioned beneath the saddle along with a 5 mm thick layer (Prolite^TM^ half pad). Girth design and features have been described elsewhere [18]. In brief, an anatomically shaped girth, not featuring any elastic, was used throughout.

### 2.4 Kinematics–inertial measurement units

**Fig 1:**
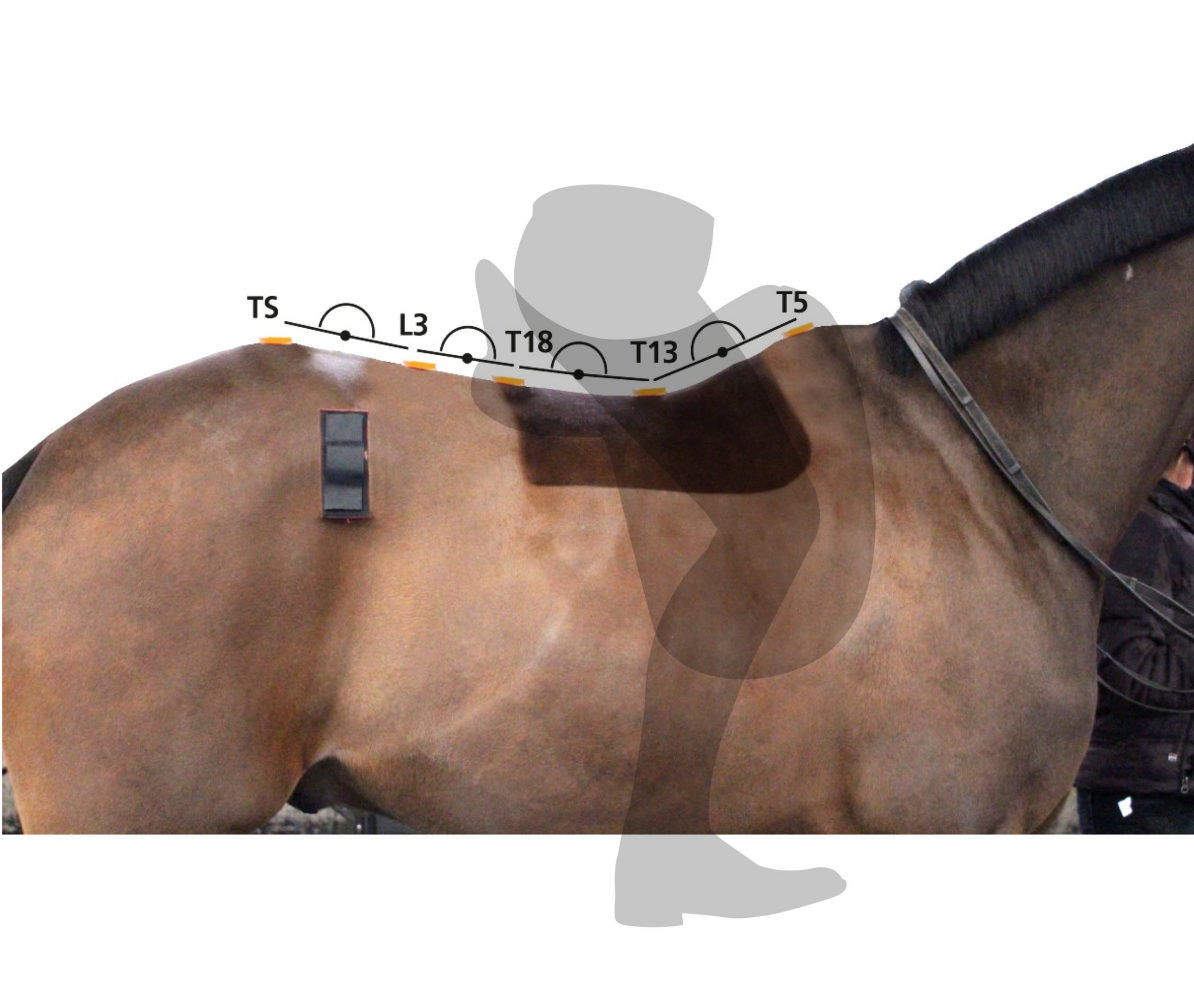
IMU sensor locations in the thoracolumbosacral region. Sensors were glued on to the skin at T5, T13, T18, L3 and TS. Sensors were positioned on the midline with sensors T5, T13 and T18 being positioned beneath the saddle. Sensor pouches were used for the left and right tuber coxae. Differential pitch, heading and roll rotations were calculated between adjacent sensors T5-T13, T13-T18, T18-L3 and L3-TS. Movement symmetry values were calculated from displacement minima and maxima for each sensor.

Horses were instrumented with eight MTw inertial measurement units (IMU) (Xsens MTw Awinda, The Netherlands) as part of a validated sensor-based system [16, 19]. Identifying landmarks by manual palpation, the same researcher (RMG), attached sensors over the poll, withers (T5), vertebral segments of the thirteenth (T13) and eighteenth (T18) thoracic vertebrae, the third lumbar (L3) vertebra, between the tubera sacrale (TS), and over the left and right tuber coxae. T5, T13, T18, L3 and TS sensors were glued on to the skin using hair extension glue (Salon Pro, London); the remaining sensors (poll & tuber coxae) were attached using custom-made pouches and double-sided tape (Fig 1). Sensor locations were referenced with white skin paint. Due to saddle design, sensors which were positioned beneath the saddle did not come into contact with the medial margins of the saddle panel. To reduce variability, sensors remained on the horse throughout data collection. Sensor data were collected at 60 Hz per individual sensor channel and transmitted via a proprietary wireless data transmission protocol (Xsens) to a receiver station (Awinda, Xsens) connected to a laptop computer running MTManager (Xsens) software. IMU manufacturer specifications: internal sampling rate 1000 Hz; buffer time up to 30 seconds; dimensions 47x30x13 mm; mass 16 grams; operating temperature range 0C – 50°C; and dynamic accuracy 0.75 degrees root mean square (RMS) (roll/pitch) and 1.5 degrees RMS (heading).

IMU data were processed following published protocols [16]. In brief, tri-axial sensor acceleration data were rotated into a gravity (z: vertical, positive up) and horse-based (x: craniocaudal, positive forwards and y: mediolateral, positive to the left) right-handed reference frame and numerically double integrated to displacement. Displacement data were segmented into individual strides based on vertical velocity of the sacrum sensor [20], and median values for the following kinematic variables were calculated over all strides for each exercise condition.

**Fig 2:**
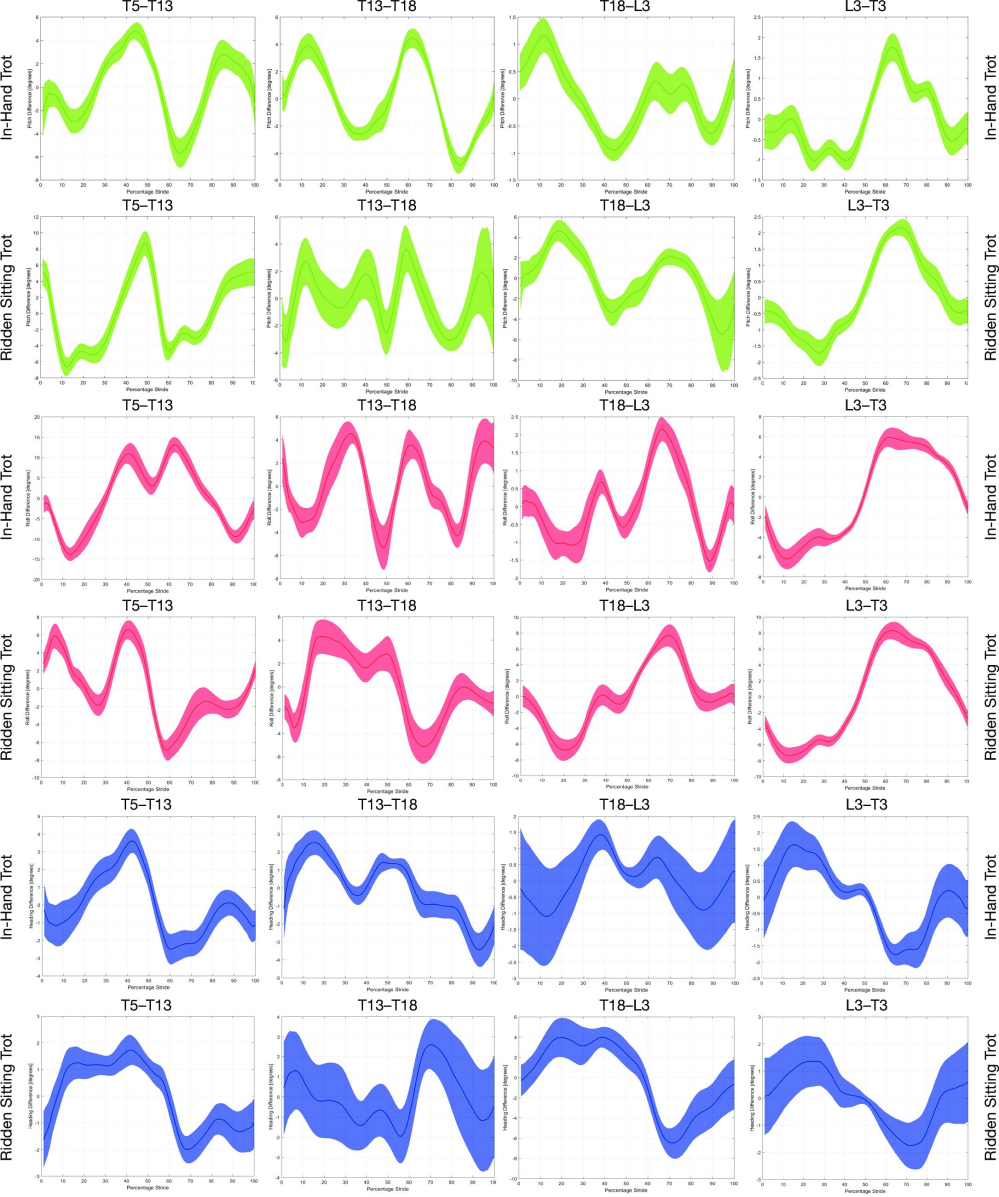
Time graphs featuring two adjacent skin mounted IMUs positioned along the thoracolumbosacral region. Time graphs for one horse displaying the differential rotational signals calculated from two adjacent skin mounted IMUs, positioned along the thoracolumbosacral region with the horse trotting in-hand and when in sitting trot. Graph ID: green = differential pitch, red = differential roll and blue = differential heading.

Orientation-time signals for roll, pitch and yaw of T5, T13, T18, L3 and TS were used to calculate differential rotational movement by subtracting signals of adjacent sensors from each other (T5-T13, T13-T18, T18-L3, L3-TS) to quantify flexion-extension (differential pitch, rotation around transverse (lateral-lateral) axis), axial rotation (differential roll, rotation around longitudinal (craniocaudal) axis) and lateral bending (differential yaw, rotation around vertical axis) of the thoracolumbar spine in degrees similar to the method introduced in [21] for flexion-extension (Fig 2).

Outcome parameters:

Differential rotational values

Differential pitch roll and heading values for T5-T13, T13-T18, T18-L3 and L3-TS for both conditions (in-hand trot and sitting trot).

Symmetry variables:

- minimum difference (D) Head (HDMin), Withers (WD_Min_), T13D_Min_, T18D_Min_, L3D_Min_ and Pelvis (PDMin): difference between the two minima in vertical (z) displacement observed during each of the two diagonal stance phases in trot.
- maximum difference (D) Head (HDMin), Withers (WD_Max_) T13D_Max_, T18D_Max_, L3D_Max_ and Pelvis (PDMax): difference between the two maxima in vertical (z) displacement observed after each of the two diagonal stance phases in trot.

Due to the lack of direct speed measurements, stride time (from IMU data) was used as a surrogate measure making use of the relationship between speed and stride time within a gait.

### 2.5 Study protocol

Warm up procedure: all horses were walked in-hand with a bridle on in both a clockwise and anticlockwise direction around the arena (20 x 60 m) for twenty minutes.

#### 2.5.1 In-hand trot data collection

IMUs were fitted to the poll and along the thoracolumbosacral regions and left/right tubera coxae. Horses were then trotted in-hand wearing a bridle. Each horse was handled from the left side and trotted in-hand by their respective handler. Horses were trotted in-hand, with their head and neck in an unrestrained position (head and neck positions (HNPs) 1, [22]). Data were collected from eleven repeated straight-line strides with three repeats totalling mean±SD 33±3 strides used for the analysis.

#### 2.5.2 Ridden data collection

Horses were prepared for ridden exercise with the fitting of the saddle, girth, saddle cloth and half pad as previously described. The same bit and bridle used for the in-hand data collection was used for the ridden part of the study. Care was taken to ensure that the T5, T13 and T18 sensors were not in contact with the medial margins of the saddle panel. One SMSQSF verified sensor location visually and manually, by placing their hand beneath the pommel and palpating the lateral edges of the T13 sensor. This observation was made with and without the rider mounted. Each horse underwent a 25-minute warm up protocol self-prescribed by the rider, which included walk, rising/sitting trot and canter on both the left and right reins. Warm up also included lateral work with four half passes, shoulder in and travers (60m) in trot and canter in both directions. After the warm up period had been completed, the kinematics of the thoracolumbar spine were quantified in a straight line in collected trot, with the rider remaining seated throughout the trot motion cycle.

An experimental track (50m x 1.5m) was created in the middle of the arena using spherical cones. The arena dimensions allowed for eleven strides in trot, with both the start and end points being determined using two cones. All measurements (in-hand and ridden) were performed in an indoor (20 x 60 m) arena on a wax coated surface. The surface was groomed prior to, and in between, each horse. Horses were ridden in collected trot (on the bit) with the bridge of their nose on the vertical (HNPs 2, [22]). Three repeats in sitting trot in a straight line were collected from eleven consecutive strides with three repeats, totalling mean±SD 33±5 strides.

### 2.6 Statistical analysis

Statistical analysis was performed in SPSS (vers. 26 IBM, Armonk, USA). Four general linear mixed models were used to analyse the kinematic data (differential rotations and symmetry values), with condition (in-hand and sitting trot) and direction (straight line portion of the horse ridden on the left rein (‘left approach’) and on the right rein (‘right approach’) through the experimental area) defined as fixed factors and horse defined as random factor. Stride time was entered into the model as a covariant and added as a fixed factor. The significance level throughout was set to P≤0.05. A Bonferroni post hoc analysis was carried out to determine pairwise differences between conditions. Instead of applying the Bonferroni correction on the significance level, alpha, this study reported the Bonferroni adjusted *p*-values (*p*- values based on Fisher’s Least Significant Difference (LSD) multiplied by the number of comparisons done). This allows assessment of significance with reference to the traditional alpha of 5%, without increasing type II errors.

## 3 Results

### 3.1 Horse inclusion

From the subjective veterinary assessment, the day preceding the experiment, all horses were deemed fit to perform. From a physiotherapy perspective all horses were found to not have muscle hypertonicity or pain in the epaxial musculature of the thoracolumbosacral spine. From the objective movement asymmetry measures horses had (mean ± SD) asymmetry values (in mm): Poll Min_Diff_ 9.3 ± 8.7, Poll Max_Diff_ -2.0 ± 5.5, Pelvis Min_Diff_, 3.1 ± 2.7, Pelvis Max_Diff_ 2.9 ± 7.6 and HHD 4.9 ± 14.3.

### 3.2 Stride time

Stride time in millisecond (ms) when trotting in-hand was 802.7 ± 26.0 ms and when ridden 850.55 ± 40.4 ms (P = 0.07). No difference (P = >0.21) in stride time between directions (left approach: 848.0 ± 41.6 ms and right approach: 853.1 ± 39.2 ms) was found.

### 3.3 Differential rotational movement of the thoracolumbosacral regions

#### 3.3.1 T5-T13

Differential roll and heading values differed between conditions (P = 0.04, P = 0.01). Post hoc analysis showed an increase in roll when trotting in-hand compared to when ridden in sitting trot (in-hand 24.5° (2.3), ridden 18.2° (1.5), P = 0.04), and an increase in heading values when trotting in-hand compared to when ridden in sitting trot (in-hand 7.1° (0.5), ridden 5.2° (0.3), P = 0.01). No significant differences were found between conditions for differential pitch (P = >0.48) (Table 1).

**Table 1:** Displaying estimated marginal mean (EMM) +/- standard error (SE) for differential pitch, roll and heading rotational values (in degrees) for the thoracolumbosacral spine in the unloaded condition in-hand trot and sitting trot from 33 straight trots.

	Segment	In-Hand (IH) (Unloaded) Trot Straight	Ridden Sitting Trot (ST) Pooled	Stride Time Effect	Gait Effect	Bonferroni Post Hoc	Ridden Sitting Trot Left Approach	Ridden Sitting Trot Right Approach	Ridden Directional Effect	Stride Time Effect
		EMM SE (+/-)	EMM SE (+/-)	P Value (In-Hand–Ridden)	P Value		EMM SE (+/)	EMM SE (+/-)	P Value	P Value (left–right rein)
**Differential Pitch Rotation**	**T5-T13 (°)**	9.5 (1.1)	8.0 (0.8)	0.48	0.25	-	8.1 (1.0)	8.1 (1.1)	0.95	0.23
	**T13-T18 (°)**	9.8 (0.7)	9.7 (0.5)	0.07	0.86	-	9.6 (0.6)	10.6 (0.6)	0.16	0.21
	**T18-L3 (°)**	3.9 (0.5)	6.3 (0.3)	0.07	**<0.0001**	IH < ST, P<0.0001	7.5 (0.4)	7.3 (0.4)	0.67	0.45
	**L3-TS (°)**	3.9 (0.4)	5.1 (0.2)	0.09	**0.04**	IH < ST P = 0.04	4.9 (0.3)	4.9 (0.3)	0.92	0.37
**Differential Roll Rotation**	**T5-T13 (°)**	24.5 (2.3)	18.2 (1.5)	0.36	**0.04**	IH > ST, P = 0.04	18.8 (1.8)	18.6 (1.8)	0.82	0.36
	**T13-T18 (°)**	11.9 (1.4)	15.8 (0.9)	0.11	**0.03**	IH < ST, P = 0.03	15.9 (1.1)	14.7 (1.2)	0.29	0.53
	**T18-L3 (°)**	7.7 (1.1)	11.6 (0.9)	0.28	**0.003**	IH < ST, P = 0.003	11.9 (0.9)	12.1 (0.9)	0.84	0.53
	**L3-TS (°)**	18.6 (2.0)	18.1 (1.8)	0.12	0.71	-	18.1 (1.5)	19.2 (1.5)	0.30	0.54
**Differential Heading Rotation**	**T5-T13 (°)**	7.1 (0.5)	5.2 (0.3)	0.22	**0.01**	IH > ST, P = 0.01	5.5 (0.5)	4.7 (0.5)	0.29	0.39
	**T13-T18 (°)**	6.9 (0.6)	6.2 (0.5)	0.15	0.34	-	6.8 (0.5)	6.1 (0.5)	0.15	0.22
	**T18-L3 (°)**	4.2 (0.8)	9.5 (0.6)	0.71	**<0.0001**	IH < ST, P = <0.0001	9.4 (0.8)	9.7 (0.8)	0.70	0.93
	**L3-TS (°)**	3.0 (0.5)	4.1 (0.3)	0.83	0.14	-	4.0 (0.5)	4.1 (0.5)	0.93	0.62

Table showing directional (left versus right approach) and gait effect, stride time and outcome of Bonferroni post hoc tests (P≤0.05). Figures in bold represent significant values.

#### 3.3.2 T13-T18

Differential roll rotational values differed between conditions (P = 0.03). Post hoc analysis showed a decrease when trotting in-hand compared to ridden in sitting trot (in-hand 11.9° (1.4), ridden 15.8° (0.9), P = 0.03). No significant differences were found between conditions for differential pitch or heading (All = P>0.34) (Table 1).

#### 3.3.3 T18-L3

Differential pitch, roll and heading values differed between conditions (P = <0.0001, P = 0.003, P< = 0.0001). Post hoc analysis showed a decrease in pitch when trotting in-hand compared to ridden in sitting trot (in-hand 3.9° (0.5), ridden 6.3° (0.3), P = <0.0001), a decrease in roll when trotting in-hand compared to ridden in sitting trot (in-hand 7.7° (1.1), ridden 11.6° (0.9), P = 0.003) and a decrease in heading when trotting in-hand compared to ridden in sitting trot (in-hand 4.2° (0.8), ridden 9.5° (0.6), P = <0.0001) (Table 1).

#### 3.3.4 L3-TS

Differential pitch rotational values differed between conditions (P = 0.04). Post hoc analysis showed a decrease in pitch values when trotting in-hand compared to ridden in sitting trot (in-hand 3.9° (0.4), ridden 5.1° (0.2), P = 0.04). No significant differences were found between conditions for differential roll or heading (All = P>0.14) (Table 1).

## 4 Discussion

This study quantified rotational movements and symmetry of the thoracolumbosacral region using skin mounted IMUs in a small number of horses and riders. To the authors’ knowledge, this is the first study to quantify the kinematics and symmetry of the thoracolumbosacral spine, using skin mounted IMUs positioned beneath the saddle in elite dressage horses, ridden by their respective (elite) riders. In this manuscript, in order to delineate our external measurements based on skin-mounted IMUs, from the previously conducted measurements of the underlying bony landmarks characterizing axial rotation, flexion-extension and lateral bending of the spine [3–5], here the terms differential roll, pitch and heading are used to express the relative change in orientation between pairs of skin mounted IMUs [23].

Our findings provide evidence to support our first hypothesis, which predicted an increased range of motion in the caudal thoracic and lumbar region when a horse is ridden in sitting trot. Here, an increase in differential pitch, heading and roll was found for the caudal thoracic and lumbar region (T18-L3) in sitting trot, compared to trotting in-hand. A saddle with a dead weight has been reported to induce an overall extension of the back in both walk and trot [24]. Furthermore, De Cocq et al., (2009) [13] quantified lumbosacral kinematics in sitting and rising trot compared with trotting in-hand and measured an overall extension of the back which increased when the riders were in sitting trot. This suggests that the application of either a dead weight [24], or the dynamic weight of the rider [13], has an overall extending effect on the lumbar spine. The findings of De Cocq et al., (2009) are similar to the findings being presented here (increased differential pitch, heading and roll values in the caudal thoracic and lumbosacral regions when horses were ridden in sitting trot). Comparisons with previous studies should, however, be applied with caution; our findings using skin mounted IMUs express overall dynamic ranges of motion of rotational values, i.e. differences between maxima and minima over a stride cycle (Fig 2), rather than values of individual maximum or minimum values expressing the maximum amount of flexion or extension reached as reported in the previous studies [13, 24].

In a previous study using IMUs positioned beneath the saddle comparing trotting in-hand to rising trot, thoracolumbar flexion-extension values were presented for both the seated and standing phases of the rising trot [15]. In the seated phase, the part of the back under the seat of the rider was less mobile, showing decreased flexion-extension in the mid thoracic (T12-T16) and lumbar region (T16-L2), while the cranial thoracic spine (T6-T12) showed an increase in overall flexion-extension [15]. There are no studies quantifying rotational movements of the thoracolumbosacral region (beneath the saddle) during sitting trot. Applying the findings from the aforementioned study for the seated phase only, our findings differ, where in sitting trot (rider remaining seated throughout the motion cycle) an increase in rotational pitch was reported for the caudal thoracic and lumbar region. Rising trot and sitting trot induce different dynamics (kinematics and forces) [9, 10, 12] and it seems likely that vertebral movement and amplitudes for vertebral segments vary between these two different seating positions; therefore, future work should quantify back movement under various riding positions, sitting and rising trot and two-point position, allowing for a more comprehensive biomechanical interpretation of back movement.

We also provide evidence to partially refute our second hypothesis, which hypothesised an increased range of motion of the cranial thoracic spine when horses are ridden in sitting trot. Compared to trotting in-hand, when in sitting trot, differential pitch and roll rotational values of the cranial thoracic spine remained unchanged, however, heading rotational values were reduced. When ridden, the horse has to manage the increase in dynamic forces and movement variability from the rider and saddle [25]. The rider and saddle are positioned in the thoracic region, therefore the horse may, in order to increase the dynamic stability in this region, respond by increasing the stiffness of the spine which would result in a reduced spinal range of motion. For example, after a period of resistance band training, horses showed a decrease in mediolateral displacement and rotational ranges of motion of the thoracolumbar spine, potentially a sign of increased dynamic stability [26]. In contrast, horses ridden by an asymmetric rider showed an increase in lateral bending range of motion and mediolateral displacement of the thoracolumbar spine, potentially a sign of decreased dynamic stability [27]. It appears that the principle of ‘dynamic stability’, i.e. the amount of movement of the back in relation to the forces required for producing locomotion, involves a trade-off between stability (for efficient force production) and flexibility (for energy storage). Further quantitative evidence simultaneously measuring force and movement–and thus being able to quantify stiffness–is needed to better understand this.

When considering the cheetah, extreme flexion and extension in gallop is thought to be a locomotor adaptation, allowing for energy storage contributing to high speed and increased stride length [28]. Hence, increased rotational movement in the horse may not exclusively be a sign of decreased dynamic stability. Despite its ‘rigidity’ (smaller spinal ranges of motion), the horse is able to modulate the amount of intervertebral movement through the surrounding soft tissues [8], therefore, the increased rotational movement (differential pitch, roll and heading rotational values) observed in the caudal thoracic and lumbar segments maybe a sign of energy storage and increased locomotor forces.

The position of the head and neck is an important consideration when interpreting biomechanical data and the findings presented here. In the current study, during the unloaded condition (trot in-hand) the horse’s head and neck were unrestrained (i.e. free position (HNPs 1 [22]). Different head and neck positions affect the kinematics of the thoracolumbar spine [14, 22] and future studies should attempt to use a comparable head/neck position during in-hand and ridden exercise. The horses used in the current study were ridden in sitting trot with the bridge of their nose on the vertical and the poll the highest point (HNPs 2 [22]). It may be interesting to study the influence of different head/neck positions [22] on thoracolumbar kinematics beneath the saddle.

In the current study, differential rotations were calculated from changes in orientation of sensors attached over non-directly adjacent vertebrae, in contrast to previously reported differential rotations of directly adjacent vertebrae [3–7, 14]. This should be taken into account when comparing the results of the present study to previously reported inter-vertebral movements [3–7, 14]. Currently the sensor size (47x30x13 mm) is too large for being positioned over adjacent vertebrae, without increasing the risk of physical interference or negatively affecting wireless data transmission. Differences between rotational values could be explained by: 1) differences in measuring systems: in this study we used IMUs [15, 21, 29–32] as opposed to using skin surface or bone fixated markers [3–5]; 2) previous studies were conducted on treadmills: in the current study we chose to quantify kinematics of the thoracolumbosacral spine with horses trotting over ground [33, 34] and finally 3) the horses used in the current study were elite dressage horses trotting with collection and engagement of the hindlimb with an uphill appearance. Further work is required to determine whether there are dynamic differences in the kinematics of the thoracolumbosacral spine when comparing different horse types, breeds [11], conformation [35] and trotting stride kinematics [36].

The authors appreciate that this study is limited by its sample size and further limited by the small number of riders studied, therefore caution should be taken when generalizing the findings being presented here. Furthermore, the warm-up period was self-prescribed by the rider, and was for a length of twenty-five minutes and included lateral movements. In order to minimize the potential influence of the warm-up period on thoracolumbar ranges of motion, data collection would be required to be performed immediately after the load (rider) was applied. This was not possible due to the use of ‘elite’ horses which would not have been allowed to perform the required (collected) exercise without a warm-up period.

Future studies should quantify back movement in an unloaded state, followed by saddle only condition [11], and then a saddle + rider condition. This would differentiate between the effects of the saddle alone, and the saddle/rider combination on the kinematics of the thoracolumbosacral spine. Quantifying back movement over the warm-up period may be a useful addition to the protocol providing useful information about the ‘biomechanical’ consequences of specific warm-up protocols. Given the calibre of horses, no attempts were made to standardise speed as the horses were ridden in collected trot (compared to free trot) [37]. When stride time was entered into the mixed model, it was found not to have a significant effect on any of the kinematic variables, however, this should be considered in future study designs.

The findings of this study in a small sample of elite horses and their riders are encouraging. The method applied here enables quantitative measurement of thoracolumbosacral kinematics under practically relevant conditions, including in the ridden horse providing data from underneath the saddle. The use and application of this technology, combined with force platforms measuring ground reaction forces and additional quantitative assessment of the rider/horse interaction [38–41], creates opportunity to quantify spinal kinematics in a wider population of horses, in order to add to existing knowledge of back movement in non-lame horses [33, 42]; allowing to kick-start the building of a database that is essential for advancing our understanding about back related conditions affecting equine health and performance. This study used elite horses and riders, therefore, the changes reported here may not be directly transferable to non-elite riders, where differences in spinal kinematics may reflect different strategies to compensate for rider balance, skill and asymmetry [27].

**Fig 3:**
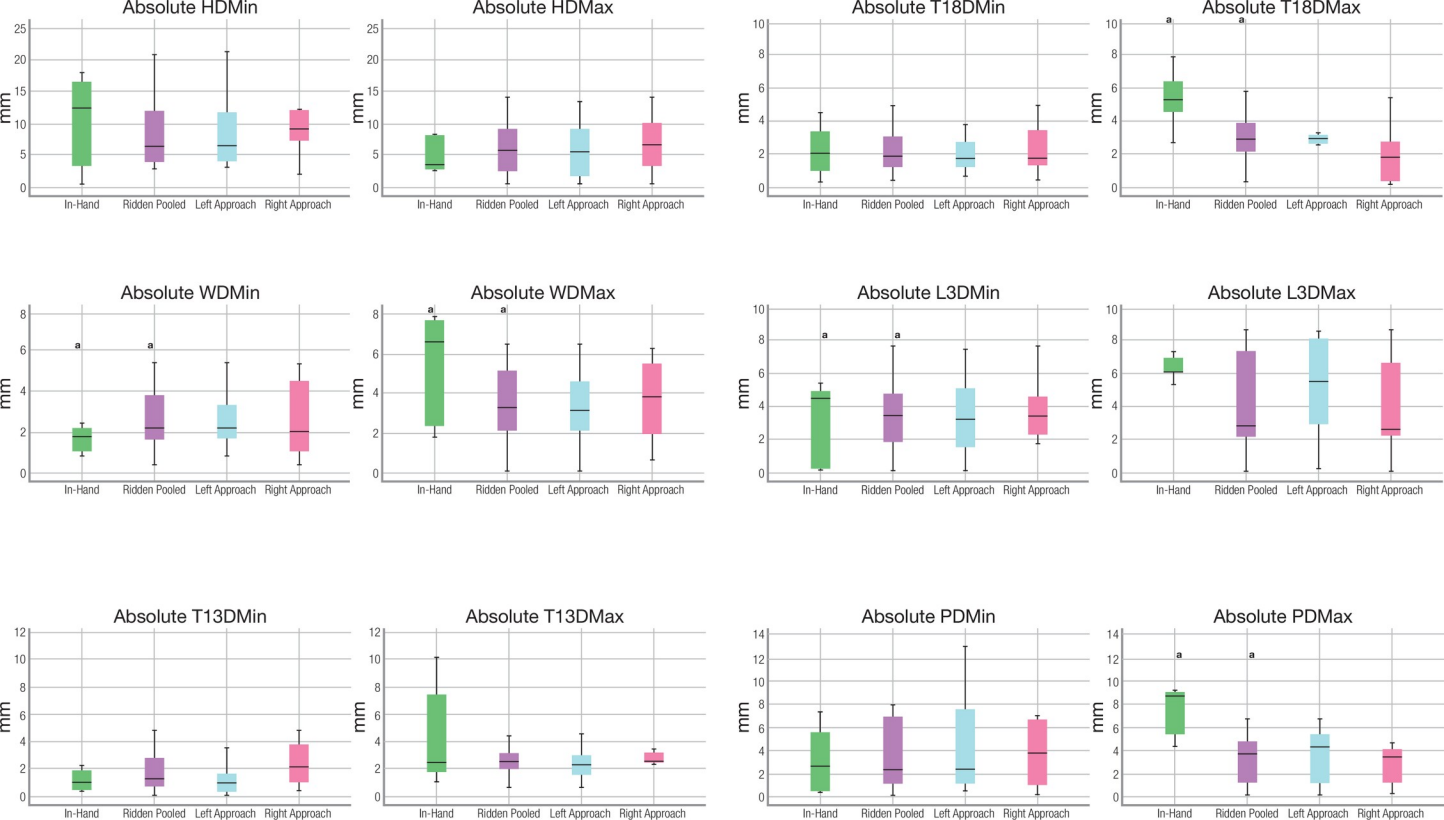
Symmetry movement values of the thoracolumbosacral regions. Boxplots displaying absolute minimum difference Head (HDMin), WD_Min_, T13D_Min_, T18D_Min_, L3D_Min_ and Pelvis (PDMin) in mm: Difference between the two minima in vertical (z) displacement observed during the two diagonal stance phases in trot and absolute maximum difference (HDMax), WD_Max_, T13D_Max_, T18D_Max_, L3D_Max_ and Pelvis (PDMax) in mm: Difference between the two maxima in vertical (z) displacement observed after the two diagonal stance phases in trot from eight horses (due to transmission technical issues, two horses data was lost). The central line represents the median; the box represents the 25th and 75th percentiles; and the whiskers represent the maxima and minima not considered to be outliers; ° represents outliers and * represents extreme outliers. ‘a’ represents the pairwise differences (P = <0.05) between conditions. When comparing the in-hand trot with sitting trot (left and right approaches pooled), absolute symmetry values were closer to zero (indicating increased symmetry) and significantly different for WD_max_ (P = 0.02) T18D_max_ (P = 0.04), L3D_min_ (P = 0.04) and PD_max_ (P = 0.01). No differences were found between the remaining parameters or when comparing left and right approaches.

Lastly this study quantified the symmetry of the thoracolumbosacral region under ridden conditions. The difference between the two minima and maxima in vertical (z) displacement observed during the two diagonal stance phases in trot were quantified for each IMU (head, withers, T13, T18, L3 and pelvis). In accordance with our third hypothesis, compared with trotting in-hand, absolute MaxDiff values closer to zero were found (representing more symmetrical movement) for the ridden exercise for T5, T13, T18 and TS (Fig 3). It is speculated that this change in symmetry during the propulsive phase, could be interpreted as the rider exerting a stabilising effect on the horse [25]. This suggestion warrants further investigation. Equine laterality, handedness and preferences are becoming better understood, especially in relation to health and athletic performance [43–51]. In the current study there were no differences in movement symmetry parameters of the thoracolumbosacral region (P = >0.10) when entering the experimental track from a left or right direction (left versus right approach). Although not significant, median absolute asymmetry values varied between left and right approach. These kinematic differences, during left and right approach, warrant future investigation.

## 5 Conclusion

This study has reported changes in the kinematics of the thoracolumbar spine when horses are trotting in a straight line and when ridden in sitting trot. When ridden, rotational values increased in the caudal thoracic and lumbar region. Differential pitch and roll appear to remain unaffected by ridden exercise in the cranial thoracic region. Symmetry values were closer to zero when ridden in sitting trot during the push off component of the stride. The method utilised here provides a means to quantify back movement and symmetry in horses, which can be used to advance our understanding of back kinematics and can help with the management of back related conditions.

## Supporting information

S1 Data (XLSX)
